# Near-infrared/glutathione-activated CRISPR/Cas13a sensing platform for the detection of multiple microRNAs

**DOI:** 10.1039/d6ra00157b

**Published:** 2026-07-02

**Authors:** Zhenhua Zeng, Ayang Wu, Haimin Gao, Yanping Zheng, Huicong Yang, Xiaojie Yao, Zhongjie Yang

**Affiliations:** a Department of Laboratory Medicine, Zhangzhou Affiliated Hospital of Fujian Medical University Zhangzhou 363000 China 13906969368@139.com 13799827493@163.com

## Abstract

MicroRNAs (miRNAs) are important liquid biopsy biomarkers, and their multiplex detection is highly important for the early screening of hepatocellular carcinoma. This work developed a CRISPR/Cas13a biosensing platform based on near-infrared (NIR) light and glutathione (GSH) dual activation for highly sensitive and specific determination of miRNA-21, miRNA-155, and miRNA-224. The advantages of this platform lie in the introduction of GSH-responsive mesoporous silica carriers and photolytic linkers (pc-linkers), which transform the sensing process from “passive triggering” to “controllable activation”, effectively suppressing nonspecific signals. Specifically, GSH can degrade mSiO_2_ with tetrasulfide bonds, thereby degrading the carrier. It will release the CRISPR/Cas13a system loaded on the carrier, thereby achieving specific recognition and cleavage of miRNA-21/155; at the same time, 808 nm NIR light excites upconversion nanoparticles (UCNPs) to generate ultraviolet light, which cleaves the pc-linker to activate the detection of miRNA-224. The detection limits of the developed assay for detecting miRNA-21, miRNA-155, and miRNA-224 were 4.2 pM, 5.7 pM, and 0.205 nM, respectively. The recoveries in the serum spiked experiments were 98.97–109.24%, with a relative standard deviation of less than 4.613%, indicating that the method has good accuracy and reliability. This work proposes a novel and reliable strategy for detecting multiple miRNAs in complex biological samples.

## Abstract 2

MicroRNAs (miRNAs) are important liquid biopsy biomarkers, and their multiplex detection is highly important for the early screening of hepatocellular carcinoma.

## Graphical abstract

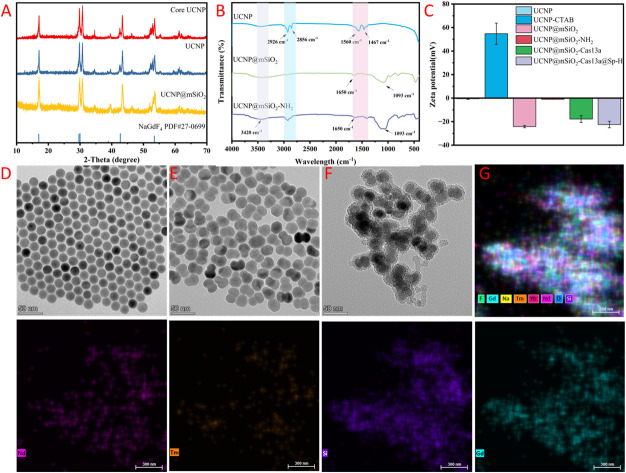

## 1 Introduction

Hepatocellular carcinoma is a common malignant tumor worldwide, and its incidence and mortality rates are increasing annually, indicating that it poses a serious threat to human health.^1–3^ Early analysis and treatment play important roles in improving cure rates, enhancing patients' quality of life, prolonging survival, and reducing medical costs.^4,5^ At present, liver tissue biopsy pathology examination is still the gold standard for early screening and diagnosis of hepatocellular carcinoma in clinical practice,^6,7^ but its invasiveness is not suitable for early universal screening of large-scale populations. In contrast, liquid biopsy has the advantages of being fast, noninvasive, highly reproducible, and cost-effective, making it more suitable for early screening and dynamic monitoring.^8^ Among numerous liquid biopsy biomarkers, microRNAs (miRNAs) are short noncoding RNAs that regulate key biological processes, including cell proliferation, differentiation, and apoptosis.^9,10^ Research has shown that the abnormal expression of multiple miRNAs (including miRNA-21, miRNA-155, and miRNA-224) is linked to the development, progression and metastasis of hepatocellular carcinoma,^11,12^ and their detection has important value for early warning and prognostic evaluation of hepatocellular carcinoma. Therefore, there is a pressing need to develop new methods that can measure these miRNAs with sensitivity and specificity.

The commonly used miRNA detection methods currently include northern blotting,^13,14^ microarray chips,^15,16^ and real-time quantitative polymerase chain reaction (RT-qPCR).^17,18^ Northern blotting is cumbersome to perform and has low sensitivity; microarray chips have high throughput but limited quantitative accuracy. Although RT-qPCR has excellent sensitivity and specificity and is known as the gold standard for nucleic acid quantification, owing to the short length of miRNA fragments, it usually requires special designs such as stem loop primers for reverse transcription, which are complex and costly. In recent years, biosensing technologies, such as electrochemiluminescence,^19,20^ photoelectrochemistry,^21,22^ and fluorescence sensing,^23,24^ have provided new avenues for miRNA detection. Among them, fluorescent biosensors are highly favorable because of their fast response, simple operation, and ease of miniaturization.^25^ However, traditional fluorescent sensors based on hairpin probes such as “molecular beacons” are often in an “always on” state, which is susceptible to nonspecific interference in complex biological sample matrices and leads to nontargeted ring opening, resulting in elevated background signals and false positive results, limiting their detection accuracy and practical application reliability. Therefore, developing a novel fluorescence sensing system with controllable activation characteristics to reduce background interference and enhance detection specificity has become a pressing challenge in this field.

The unique optical properties of lanthanide-doped upconversion nanoparticles (UCNPs) provide innovative solutions to the abovementioned problems.^26^ Unlike traditional downconversion luminescent materials such as organic dyes and quantum dots, UCNPs can emit higher-energy visible light and even ultraviolet light through multiphoton processes under near-infrared light (NIR, such as 808 nm) excitation, known as anti-Stokes luminescence.^27^ This feature has two significant advantages: first, NIR light greatly avoids fluorescence interference from biological samples themselves; second, UCNPs have good luminescence stability and strong resistance to photobleaching. Therefore, UCNPs are often used as excellent energy donors for constructing high signal-to-noise ratio fluorescent biosensors.^28^ Notably, through their reasonable design, UCNPs can utilize the ultraviolet light generated under NIR excitation to cleave chemical bonds sensitive to ultraviolet light, such as photocleaving bonds and photolytic linkers (pc-linkers), thereby achieving “photocontrolled” activation of the detection process and shifting the sensing mode from “passive triggering” to “on-demand activation”, effectively avoiding nonspecific signals. In addition, the use of glutathione (GSH) to reduce broken disulfide bonds is another effective chemical strategy for achieving “controllable activation”.^29^ However, research based on NIR and GSH dual-activation sensors is few. In addition, miRNAs are characterized by short fragments, low abundance, and multiple homologous sequences,^30^ which present challenges in achieving high-sensitivity and high-specificity detection.

The emergence of clustered short palindromic repeat sequences and their associated protein (CRISPR/Cas) systems with regular intervals provides a powerful tool for highly specific nucleic acid recognition and signal amplification.^31^ Among them, the CRISPR/Cas13a system can be activated in the presence of RNA targets, which exhibit nonspecific *trans*-cleavage activity and are capable of cleaving single-stranded RNA reporter molecules.^32^ The reaction can be carried out at a constant temperature (such as 37 °C) without the need for complex thermal cycling equipment. These characteristics indicate that CRISPR/Cas13a holds significant promise for molecular diagnostics.^33^ However, current research on miRNA detection *via* CRISPR/Cas13a is relatively limited and has focused mostly on a single target. In the early diagnosis of tumors, the combined detection of multiple biomarkers often reflects the heterogeneity of the disease more comprehensively than a single biomarker does, significantly improving the sensitivity and specificity of diagnosis.^34^ Therefore, developing a sensing platform that can combine advanced nanomaterials (such as UCNPs) with efficient molecular tools (such as CRISPR/Cas13a) and integrate multiple “controllable activation” modes to sensitively and specifically detect multiple miRNA targets has important scientific significance and application value.

**Scheme 1:**
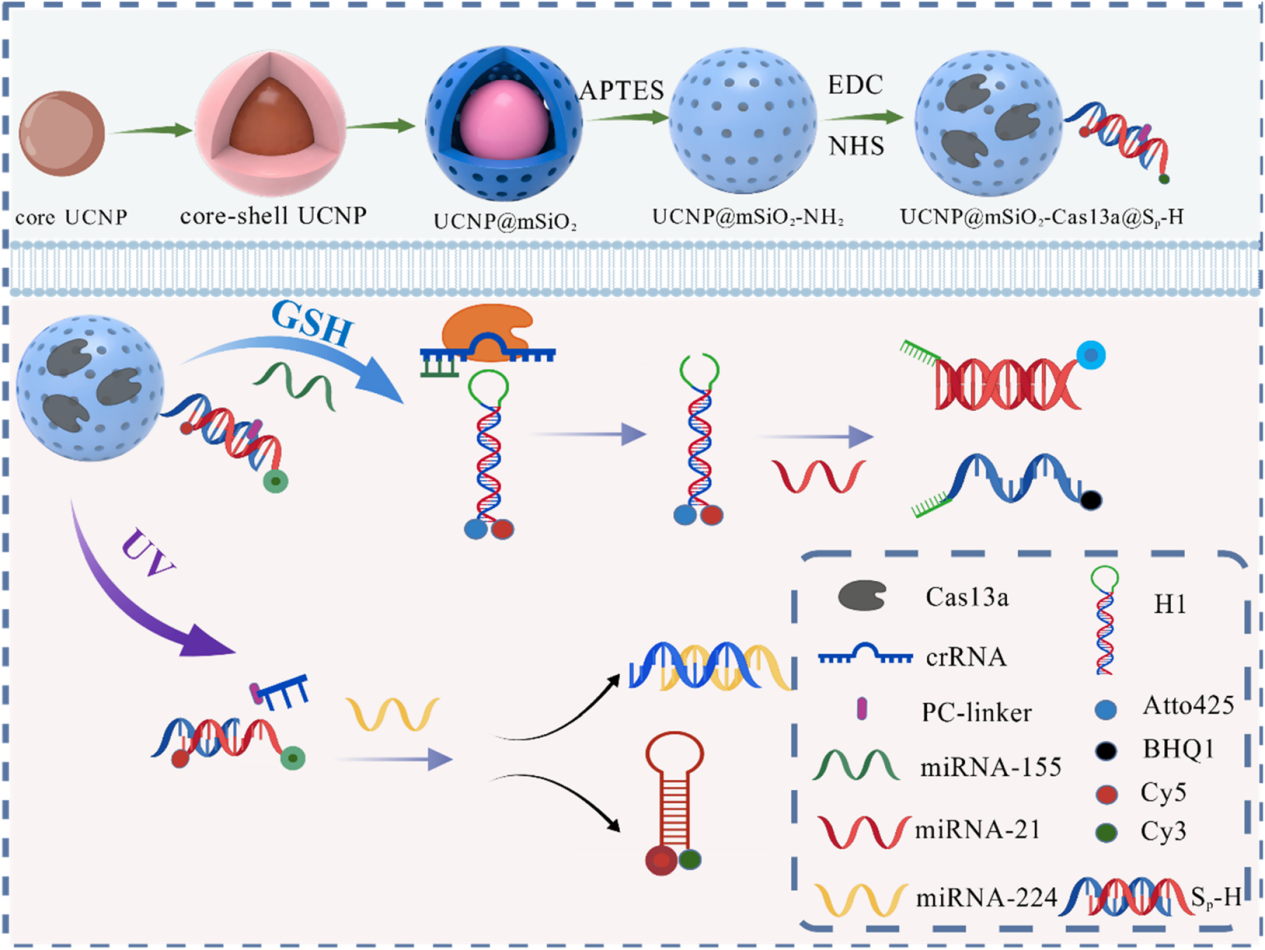
NIR/GSH dual-activation biosensing platform for detecting miRNAs.

This work developed a CRISPR/Cas13a sensing system with dual activation of NIR and GSH for high-performance determination of miRNA-21, miRNA-155, and miRNA-224. The core goal of this work is to transform the “passive triggering” mode of traditional sensors into a “controllable activation” mode by introducing mesoporous silica nanocarriers responsive to GSH degradation and pc-linkers controlled by NIR light, thereby constructing an NIR/GSH dual-activation biosensing platform. The specific principle is as follows (Scheme 1): on the one hand, in the presence of glutathione, tetrasulfide bonds are broken. As a result, the mesoporous silica structure bridged by tetrasulfide bonds will degrade, causing the carrier structure to collapse and release the CRISPR/Cas13a complex it has loaded. When the target miRNA-21 and miRNA-155 are present, the *trans*-cleavage activity of Cas13a is specifically activated, cleaving the single-stranded RNA reporter molecule (H1) with a quenching group (BHQ1) and a fluorescent group (Atto425), leading to fluorescence recovery and detection of these two miRNAs. On the other hand, under 808 nm irradiation, the UCNPs convert excitation light to ultraviolet light, cleaving the pc-linker in the double-stranded DNA structure (Sp-H) and thereby exposing the specific binding site of miRNA-224. Subsequently, miRNA-224 binds to the Sp chain through a chain displacement reaction, and the released H chain spontaneously forms a hairpin structure. Owing to the labelling of the fluorescent donor Cy3 and acceptor Cy5 on both ends of the hairpin, significant fluorescence enhancement signals were generated at 660 nm through fluorescence resonance energy transfer at the excitation wavelength of the donor (525 nm), resulting in the detection of miRNA-224. This controllable activation mechanism effectively suppresses nonspecific reactions in the absence of the target, significantly reducing background signals. This study not only presents a new strategy for highly sensitive and specific determination of low-abundance miRNAs in complex biological samples but also offers potential tools for early screening and subtyping diagnosis of hepatocellular carcinoma.

## 2 Experimental

### 2.1 Instruments

The material morphology was characterized *via* a Talos F200i high-resolution transmission electron microscope (TEM) at 200 kV, with concurrent elemental analysis by energy-dispersive spectroscopy (EDS). The crystal structure of the sample was analyzed by X-ray diffraction (XRD) on a MiniFlex-600 diffractometer using Cu Kα radiation, with a 2*θ* range of 10°–80° at a scan rate of 10° min^−1^. Zeta potential was assessed using a Nano-ZS90 system. Fourier transform infrared (FT-IR) spectroscopy was performed using a Nicolet 5700 spectrometer. Electrophoresis and subsequent gel imaging were performed using a Luna II system. Ultraviolet-visible (UV-vis) spectra were collected with a LAMBDA 950 spectrophotometer. The fluorescence properties were characterized by recording emission and excitation spectra on a FluoroMax-4 spectrofluorometer. Under excitation with a 980 nm CW laser (1 W), the upconversion luminescence spectra were recorded.

### 2.2 Agents

Rare earth chlorides, oleic acid (OA), 1-octadecene (ODE), NH_4_F, sodium citrate, anhydrous ethanol, cyclohexane, methanol, hexadecyltrimethylammonium bromide (CTAB), bis[3-(triethoxysilyl)propyl]tetrasulfide (BTESPT), tetraethyl orthosilicate (TEOS), γ-aminopropyltriethoxysilane (APTES) and NaOH were sourced from Aladdin Chemical Reagent Co., Ltd. 1-(3-Dimethylaminopropyl)-3-ethylcarbodiimide hydrochloride (EDC), *N*-hydroxysuccinimide (NHS), Tris + EDTA (TE) buffer (1×, pH = 8) and HEPES Buffer Solution (HEPES) were purchased from Sangon Biotechnology. All the DNA strands used in the experiments were provided by Sangon Biotechnology. The oligonucleotide sequences are provided below (5′ → 3′):

Sp: COOH-GGAACGGAACCACTAGTGACTTG/iPC-linker/GTTGACC

H: TGGTCAACTGGAGTACTAGTGGTTCCGTTCCA

H1: Atto425-CGTCAACATCAGTCTGATAAGCTA/rU//rU//rU//rU//rU//rU/CTGCTCAGACTGATGTTGACG-BHQ1

miRNA-21: UAGCUUAUCAGACUGAUGUUGA

miRNA-224: CAAGTCACTAGTGGTTCCGTT

miRNA-155: UUAAUGCUAAUCGUGAUAGGGGU

miRNA-122: UGGAGUGUGACAAUGGUGUUUG

miRNA-222: AGCUACAUCUGGCUACUGGGU

miRNA-221: AGCUACAUUGUCUGCUGGGUUUC.

### 2.3 Preparation and modification of UCNP

#### 2.3.1 Preparation of core UCNP

The core NaGdF_4_:Yb,Tm of the upconversion nanoparticles was prepared using the solvothermal method.^35^ First, GdCl_3_·6H_2_O (0.30 mmol), TmCl_3_·6H_2_O (0.01 mmol), and YbCl_3_·6H_2_O (0.69 mmol) were combined. Then, 6 mL of OA and 15 mL of ODE were added, and the mixture was stirred thoroughly. The solution was heated to 160 °C until the powder dissolved. After cooling to room temperature, 10 mL of a methanol mixture containing NaOH and NH_4_F was added, and the mixture was allowed to react for 30 min. The mixture was then heated to 300 °C and allowed to react for 60 min. The reaction mixture was allowed to cool to room temperature upon completion, followed by centrifugation, precipitation, and washing to afford the NaGdF_4_:Yb,Tm product.

#### 2.3.2 Preparation of core–shell UCNPs

GdCl_3_·6H_2_O (0.48 mmol), NdCl_3_·6H_2_O (0.24 mmol) and YbCl_3_·6H_2_O (0.08 mmol) were combined. 6 mL of OA and 15 mL of ODE were added and mixed thoroughly. The solution was heated to 160 °C until the powder dissolved. Following the addition of the NaGdF_4_:Yb,Tm nanoparticles at room temperature, the mixture was heated to 110 °C for 30 min and then cooled. A 10 mL methanol solution containing NaOH and NH_4_F was added. After reacting for 30 min the mixture was heated to 300 °C for 60 min. Upon completion of the reaction, the mixture was cooled to room temperature. The core–shell nanoparticles were then collected by centrifugation and purified through successive cycles of precipitation and washing.

#### 2.3.3 Modification of core–shell UCNP

First, 0.1 g of CTAB was dissolved in 20 mL of distilled water. After ultrasonic magnetic stirring, a clear and transparent solution formed. The abovementioned cyclohexane-dispersed UCNP (5 mg mL^−1^) was added dropwise to the CTAB solution. Magnetic stirring was carried out at room temperature for 24 h, and the cyclohexane was evaporated to form the stable UCNP of CTAB, namely, CTAB-UCNP.

### 2.4 Preparation of UCNP@mSiO_2_

20 mL of distilled water and 3 mL of ethanol were mixed evenly with magnetic stirring in a flask. First, 10 mL of the CTAB-UCNP aqueous solution mentioned above was slowly added to a mixed solution of ethanol and water under magnetic stirring (750 rpm). The pH of the system was adjusted with NaOH solution to 11, and the temperature was increased to 70 °C. 200 µL (140 µL of TEOS and 60 µL of BTESPT) were slowly added to the bottle, and vigorous magnetic stirring was maintained at 70 °C for 2 h. After cooling to room temperature, the mixture was subsequently centrifuged, washed, and dried to obtain UCNP@mSiO_2_.

### 2.5 Construction of fluorescent biosensors

The above 60 mg of UCNP@mSiO_2_ was added to 30 mL of ethanol solution, 150 µL of APTES and 150 µL of deionized water. The mixture was heated to 45 °C and stirred for 8 h. The product was isolated by centrifugation and washed three times with deionized water. The resulting UCNP@mSiO_2_–NH_2_ nanoparticles were placed in a vacuum drying oven and dried for 12 h.

The resultant UCNP@mSiO_2_–NH_2_ was added to 200 µL of HEPES buffer (125 mM HEPES, 685 mM NaCl, pH 7.4). Cas13a and crRNA were added, and the mixture was stirred at room temperature for 12 h. The mixture was subsequently centrifuged and washed to obtain UCNP@mSiO_2_–Cas13a.

Then, 10 µL of Sp-H was added to 2 mL of PBS, 5 mg of EDC and 10 mg of NHS. The mixture was stirred for 2 h. The prepared UCNP@mSiO_2_–Cas13a nanoparticles were added, and the mixture was stirred overnight. The product was collected by centrifugation to obtain UCNP@mSiO_2_–Cas13a@Sp-H.

### 2.6 Fluorescence detection of miRNA-21, miRNA-155, and miRNA-224

#### 2.6.1 Detection of miRNA-21 and miRNA-155

Appropriate amounts of prepared UCNP@mSiO_2_–Cas13a@Sp-H dispersion; 6 µL of GSH(10 mM) solution; different concentrations of target miRNA-21 (0.5, 2.5, 5, 10, 15, 20, 25, 30, 35 and 40 nM); and miRNA-155 (0.25, 1.25, 5, 7.5 and 10 nM) were mixed together with a total volume of 200 µL. The reaction proceeded at 37 °C for 120 min. After incubation, the fluorescence emission spectra of the solution were collected by a fluorescence spectrometer in the wavelength range of 440–600 nm.

#### 2.6.2 Detection of miRNA-224

An equal amount of the UCNP@mSiO_2_–Cas13a@Sp-H dispersion was irradiated with an 808 nm laser for 16 min. Subsequently, different concentrations of the target miRNA-224 solution were added to reach final concentrations of 2.5, 5, 10, 15 and 20 nM with a total volume of 200 µL. The reaction system was maintained at 37 °C for 120 min. After incubation, the fluorescence emission spectra of the solution were collected by a fluorescence spectrometer in the wavelength range of 440–600 nm. All experiments were independently repeated three times to ensure the reliability of the data.

### 2.7 Detection of miRNAs in actual serum samples

To evaluate the detection performance of the constructed sensor in actual complex samples, human serum was selected as the matrix for spiked recovery experiments. First, to reduce matrix interference, the original serum sample was diluted 100 times with PBS. The standard addition assay was subsequently used for detection: a series of known concentrations of miRNA-21, miRNA-155, and miRNA-224 standards were accurately added to the diluted serum sample, after which the operation and fluorescence determination were carried out according to the steps described in Section 2.6. To verify the accuracy and feasibility of this method in actual sample analysis, the recovery rate of the target miRNA was calculated.

## 3 Results and discussion

### 3.1 Preparation and characterization of the nanomaterials

The aim of this work is to construct a “controllable activation” fluorescent biosensor whose activation mechanism depends on two responses: one is the ultraviolet light generated by the UCNP under NIR light excitation, which is used to cleave photosensitive chemical bonds; the second is the use of GSH to reduce and break disulfide bonds. Based on this design, the core UCNP was first synthetized using the solvothermal approach, and the core–shell structure UCNP was obtained by epitaxial growth. Then, the phase transfer of UCNP was carried out with the cationic surfactant CTAB, and with it as the core, mSiO_2_ was coated by the sol–gel method to obtain UCNP@mSiO_2_. This mesoporous structure provides a carrier for loading Cas13a. Next, APTES was used to aminate the surface of the silica, which was then coupled with reporter DNA (Sp-H) *via* an acylation reaction to construct the UCNP@mSiO_2_–Cas13a@Sp-H sensing platform.

**Fig. 1:**
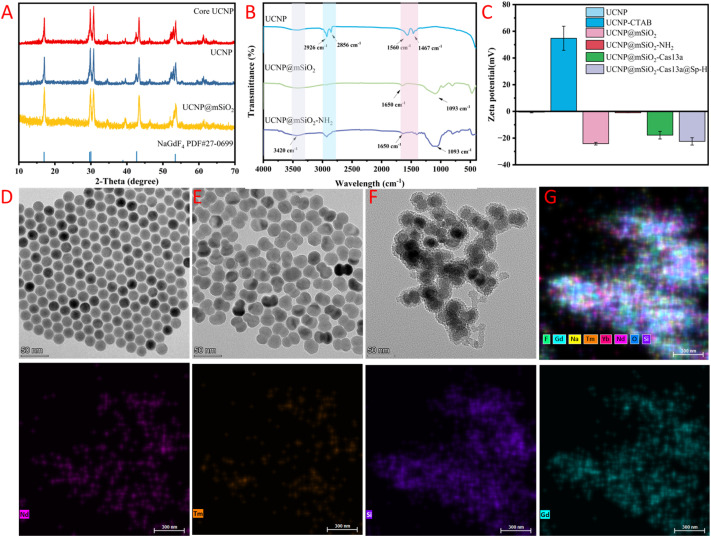
(A) XRD pattern of the synthesized nanomaterial; (B) FT-IR spectroscopy of the nanomaterials; (C) zeta potential of the nanomaterials at different modification stages; (D) TEM image of core UCNP; (E) TEM image of core–shell UCNP; (F) TEM image of UCNP@mSiO_2_; (G) EDS analysis spectrum of UCNP@mSiO_2_.

The morphology and structure of the obtained nanomaterials were characterized using XRD, FTIR, zeta potential, TEM, and EDS. As presented in Fig. 1A, the diffraction peaks (2*θ*) of NaGdF_4_:Yb,Tm and NaGdF_4_:Yb,Tm@NaGdF_4_:Yb,Nd coincided with the characteristic peaks of hexagonal NaGdF_4_ (17.005°, 29.655°, 30.002°, 42.717°, 52.88° and 53.446°), and no impurities were detected, indicating the synthesis of UCNP with good crystallinity. After mSiO_2_ coating, the characteristic peak position of NaGdF_4_ did not change obviously, indicating that the coating process did not alter the crystal structure of UCNP and that the core still maintained high crystallinity. In addition, the main diffraction peak positions of the three samples are basically consistent, further indicating that the introduction of mSiO_2_ did not affect the lattice parameters of the core. In the XRD spectrum of UCNP@mSiO_2_, a broadened diffraction peak appears at approximately 22.5°, corresponding to amorphous silica, which confirms the coating of a mSiO_2_ shell on the surface of UCNP.

Further characterization of the prepared UCNP, UCNP@mSiO_2_ and UCNP@mSiO_2_–NH_2_ was carried out using FTIR. As shown in Fig. 1B, the absorption peaks of UCNP at 1560 cm^−1^ and 1467 cm^−1^ are attributed to the stretching vibration of carboxyl groups in the surface OA ligand, while the peaks at 2856 cm^−1^ and 2926 cm^−1^ correspond to the stretching vibration of –CH_2_ in the OA molecule. After coating with mesoporous silica, an asymmetric vibration band of Si–O–Si presented at 1093 cm^−1^, and the peak at 1650 cm^−1^ originated from the stretching vibration of O–H on the surface of the silica, indicating that the mSiO_2_ had been coated on the surface of the UCNP. After silane modification, a clear N–H stretching vibration peak can be observed at 3420 cm^−1^, indicating that the amino group has been introduced into the outer layer of mesoporous silica, laying the foundation for the subsequent coupling of Sp-H through the acylation reaction.

Fig. 1C shows the zeta potential results of UCNP, UCNP-CTAB, UCNP@mSiO_2_, UCNP@mSiO_2_–NH_2_, UCNP@mSiO_2_–Cas13a and UCNP@mSiO_2_–Cas13a@Sp-H. After CTAB modification, due to its cationic surfactant properties, the surface potential significantly increased to +54 mV. After coating with mesoporous silica, the surface silicon hydroxyl groups ionized to produce SiO^−^, reducing the potential to −24.16 mV. After amino modification, the potential rebounded to −1 mV, indicating that the amino group partially replaced the surface hydroxyl group. After the Cas13a protein was adsorbed, the potential further decreased to −17.8 mV due to the negative charge on the protein surface. Finally, Sp-H was coupled, and due to the negative charge of the phosphate groups on the DNA strand, the potential continued to decrease to −22.47 mV. This series of regular changes in zeta potential gradually verified the successful modification of various functional groups and confirmed the effective construction of the detection sensing platform.

TEM images displayed good dispersion of the core UCNP, with an average size of approximately 19.14 nm (Fig. 1D). After further coating with the NaGdF_4_ shell, the resulting core–shell UCNP exhibited a dumbbell-shaped morphology, with an average size increase to 29.73 nm (Fig. 1E). The TEM image of UCNP@mSiO_2_ (Fig. 1F) clearly shows a core–shell structure, indicating that the UCNP is uniformly coated with a layer of mesoporous silica on the outside. Finally, the presence of elements such as Nd, Tm, Si, and Gd in the material was confirmed through EDS analysis (Fig. 1G). The above results collectively indicate the successful synthesis of core UCNP, core–shell UCNP, and UCNP@mSiO_2_ materials with regular morphologies and clear structures.

### 3.2 Detection principle and feasibility of the constructed sensor

**Fig. 2:**
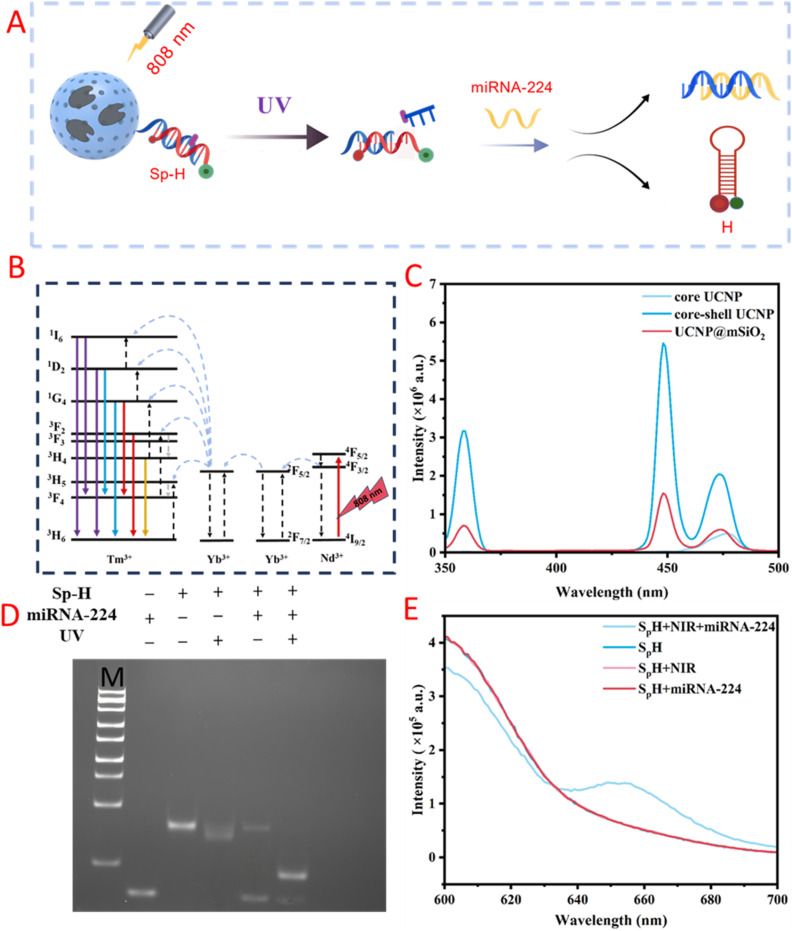
(A) Schematic diagram of the miRNA-224 detection principle; (B) luminescence mechanism of UCNP; (C) fluorescence spectra of different nanomaterials; (D) polyacrylamide gel electrophoresis; (E) fluorescence response of different detection systems at 660 nm in the presence of miRNA-224 (100 nM).

This work developed a CRISPR/Cas13a sensing system with dual activation of NIR and GSH for detecting miRNA-21, miRNA-155, and miRNA-224. In this system, UCNP is irradiated with 808 nm NIR light, and its sensitized ions absorb low-energy photons and gradually transfer energy to luminescent ions (Fig. 2B). After multiphoton absorption and energy level transition, the luminescent ions subsequently release ultraviolet light through radiative transition (Fig. 2C), which can cleave the pc linker in the double-stranded Sp-H structure, thereby initiating the detection of miRNA-224 (Fig. 2A).

To evaluate the feasibility of this sensing platform for detecting miRNA-224, polyacrylamide gel electrophoresis analysis (Fig. 2D) was carried out first. Lanes 1–5 are: miRNA-224, Sp-H, Sp-H + UV, miRNA-224 + Sp-H, and miRNA-224 + Sp-H + UV. The results showed that the Sp-H in lane 3, which was irradiated with ultraviolet light, experienced a band shift due to linker breakage. When lane 4 was not irradiated with ultraviolet light, miRNA-224 did not bind to Sp-H, and two bands representing miRNA-224 and Sp-H could be observed in the system. Lane 5 displayed a shallow band that was consistent with the migration position of Sp-H after breakage, corresponding to the double-stranded structure between miRNA-224 and Sp chains, while the other band corresponded to the H chain that self-folds into a hairpin structure.

The detection performance under different conditions was further investigated through fluorescence analysis (Fig. 2E). The results showed that, only in the presence of NIR light and the target miRNA-224 can the chain displacement reaction triggered by pc linker breakage restore the Cy5 red fluorescence signal. When lacking NIR light or target, the pc linker remains intact, the chain displacement reaction cannot be initiated, and Cy5 fluorescence is almost undetectable. The above gel electrophoresis and fluorescence analysis results jointly confirmed the design feasibility of the sensor platform for miRNA-224 detection.

**Fig. 3:**
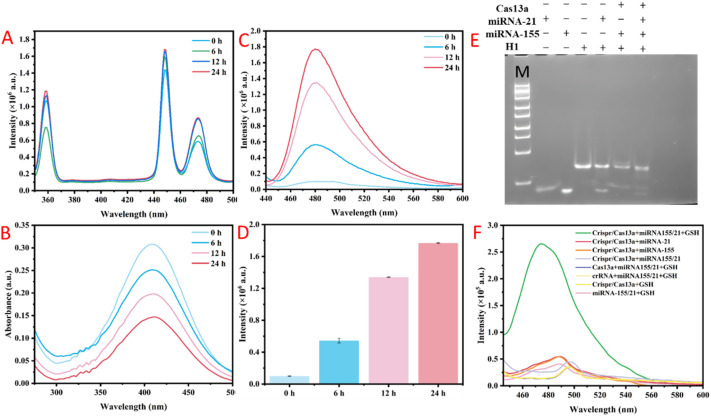
(A) Upconversion luminescence spectra of UCNP@mSiO_2_ and GSH after different reaction times; (B) UV-visible absorption spectroscopy for the DTNB method for monitoring GSH consumption in the presence of 10 mM GSH for different durations; (C and D) in the presence of GSH (10 mM), the fluorescence intensities of the molecular cleavage products corresponding to wavelengths of 475 nm at different treatment times in the presence of miRNA-21 and miRNA-155 (100 nM), *n* = 3; (E) polyacrylamide gel electrophoresis analysis; (F) in the presence of GSH (10 mM), fluorescence response of different detection systems at 475 nm in the presence of miRNA-21 and miRNA-155 (100 nM).

In this sensing system, GSH can degrade the mesoporous silica shell, thereby releasing its loaded CRISPR/Cas13a complex. When the target miRNA-21 and miRNA-155 are present concurrently, the *trans*-cleavage activity of Cas13a can be specifically activated, cleaving the single-stranded RNA reporter molecule (H1) with a quenching group (BHQ1) and a fluorescent group (Atto425) to restore fluorescence, thereby enabling the detection of these two miRNAs. To verify the GSH-responsive degradation of the mesoporous silica structure, the upconversion luminescence recovery of the UCNPs@SiO_2_ and GSH after different reaction times was first monitored. As shown in Fig. 3A, with increasing reaction time, the upconversion luminescence intensity gradually recovers, which may be due to the decrease in surface nonradiative relaxation and energy loss after decomposition of the mSiO_2_ shell. Further validation of GSH consumption was conducted using the DTNB (5,5′-dithiobis(2-nitrobenzoic acid)) indicator method (Fig. 3B). DTNB can react with the thiol group of GSH to generate bright yellow TNB (5-mercapto-2-nitrobenzoic acid anion), and its characteristic absorption peak at 412 nm gradually weakens throughout the reaction, indicating that GSH is continuously consumed. The efficiency of GSH-triggered Cas13a release was subsequently evaluated by measuring the fluorescence intensity at 475 nm over time (Fig. 3C and D). As the reaction time increased, more Cas13a was released from the degraded mSiO_2_, and the cutting efficiency of the reporter molecule H1 increased, resulting in a corresponding increase in the fluorescence signal.

Next, the detection ability of the system for miRNA-21 and miRNA-155 was verified through polyacrylamide gel electrophoresis (Fig. 3E). Lanes 1–6 are miRNA-21, miRNA-155, H1, miRNA-21 + H1, Cas13a + miRNA-155 + H1, and Cas13a + miRNA-21 + miRNA-155 + H1. Lane 5 shows that when miRNA-155, Cas13a, and H1 coexist, the H1 band shifts downwards, indicating that Cas13a is activated and cleaves H1. In lane 6, the cleaved H1 portion exists in the form of a free single chain, while the other portion binds to miRNA-21, and its band position is similar to that of cleaved H1, confirming the occurrence of cleavage and binding events under the coexistence of dual targets. The detection performance was further evaluated through fluorescence analysis (Fig. 3F). Only when Cas13a, crRNA, GSH, and H1 are present and when miRNA-21 and miRNA-155 are added concurrently, can the CRISPR/Cas13a system be activated to cleave H1 and produce an Atto425 fluorescence signal at 475 nm. If there is only a single target or no target, the system cannot be activated, and the fluorescence signal is extremely low. In addition, the coexistence of crRNA and Cas13a is necessary for the formation of an active cleavage complex, while GSH is responsible for releasing the encapsulated Cas13a, thereby achieving cleavage of the reporter molecule.

### 3.3 Optimization of detection conditions

**Fig. 4:**
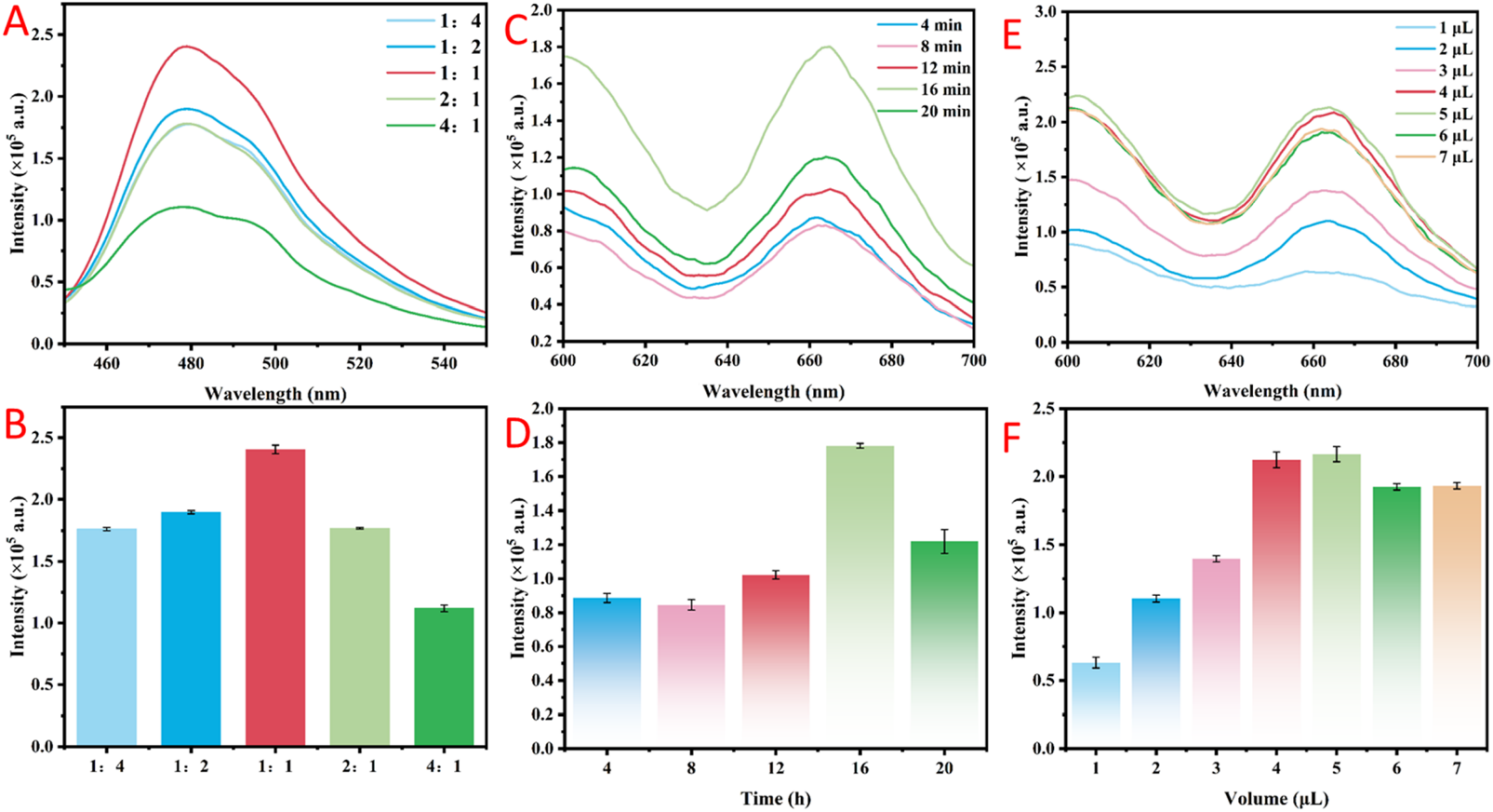
(A and B) In the presence of miRNA-21 and miRNA-155 (100 nM), fluorescence spectra of the Cas13a protein and crRNA reaction system at different molar mass ratios (Atto425, 480 nm), *n* = 3; (C and D) in the presence of miRNA-224 (100 nM), fluorescence spectra of the system under different 808 nm laser irradiation times (Cy5, 660 nm), *n* = 3; (E and F) in the presence of miRNA-224 (100 nM), fluorescence spectra of the system with various amounts of Sp-H probe added (Cy5, 660 nm), *n* = 3.

To construct an efficient fluorescence recovery detection system, we optimized key parameters, such as the molar mass ratio of the Cas13a protein to crRNA in the CRISPR/Cas13a system, the 808 nm laser irradiation time and the Sp-H dosage. First, the cutting efficiency of the Cas13a protein and crRNA at different concentration ratios was investigated. As presented in Fig. 4A and B, when the concentration ratio of Cas13a to crRNA was 1 : 1, the fluorescence recovery signal of Atto425 at 480 nm was the most significant, indicating the highest cutting efficiency at this time. Therefore, subsequent experiments adopted a 1 : 1 ratio as the optimal concentration ratio of Cas13a protein to crRNA in the CRISPR/Cas13a system. Second, the effect of the 808 nm laser irradiation time (4–20 min) on the fluorescence intensity of Cy5 at 660 nm was studied. As shown in Fig. 4C and D, with prolonged irradiation time, the fluorescence intensity of Cy5 gradually increased and reached its maximum value at 16 min. Therefore, 16 min was chosen as the optimal irradiation time. In addition, the dosage of Sp-H coupled on the UCNP@mSiO_2_ surface was further optimized. As presented in Fig. 4E and F, the fluorescence signal of Cy5 at 660 nm was strongest when 5 µL of Sp-H solution at a concentration of 1 µM was added. Therefore, this condition was determined to be the optimal dosage of Sp-H for subsequent experiments.

### 3.4 Detection performance of the constructed sensors

**Fig. 5:**
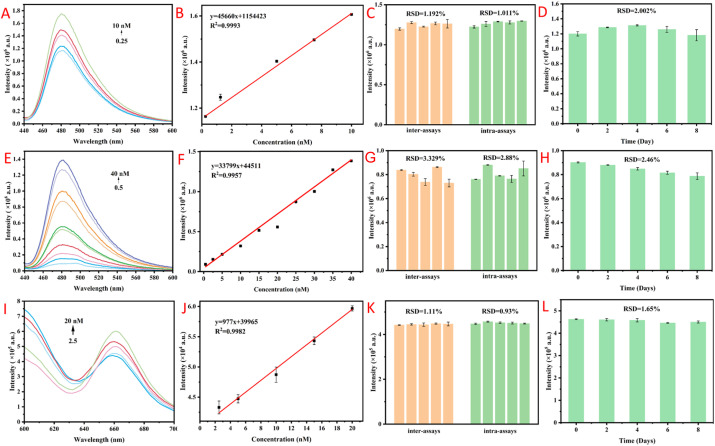
(A, E and I) Fluorescence response to different concentrations of miRNA-155/21/224, *n* = 3; (B, F and J) linear calibration curve of fluorescence intensity and the concentration of miRNA-155/21/224, *n* = 3; (C, D; G, H; K, L) repeatability and stability of the detection of miRNA-155/21/224, *n* = 3.

A systematic evaluation of the analysis performance of the proposed NIR/GSH dual-activated CRISPR–Cas13a biosensing platform was conducted under optimal experimental parameters. The fluorescence response of the biosensor to the target miRNA-155, miRNA-21, and miRNA-224 was tested by varying their concentrations. For miRNA-155, as displayed in Fig. 5A, the fluorescence intensity of the system at 480 nm gradually increased with increasing concentration. In the range of 0.25–10 nM, there was a good linear relationship between the fluorescence intensity and concentration, and the fitting equation was *y* = 45 660*x* + 1 154 423 (*R*^2^ = 0.9993; Fig. 5B). According to the formula LOD = 3*σ*/*K* (where *σ* is the standard deviation of 10 measurements of the blank sample and *K* is the slope of the calibration curve), the detection limit (LOD) is calculated to be 4.2 pM (S/N = 3). The relative standard deviations (RSDs) of the fluorescence signals for the intraday and interday repeatability tests were 1.192% and 1.011%, respectively (Fig. 5C). In addition, miRNA-155 (5 nM) was monitored continuously for 8 days, and the fluorescence signal remained stable, with an RSD of 2.002% (Fig. 5D), indicating good repeatability and stability in the determination of miRNA-155.

Similarly, for miRNA-21, the fluorescence intensity at 480 nm was linearly correlated with the concentration in the range of 0.5–40 nM (Fig. 5E and F). The fitting equation is *y* = 33 799*x* + 44 511 (*R*^2^ = 0.9957), and the calculated LOD is 5.7 pM. The RSD values for intraday, interday repeatability, and 8-day stability were 3.329%, 2.88%, and 2.46%, respectively (Fig. 5G and H). The fluorescence intensity of miRNA-224 at 660 nm was linearly correlated with its concentration in the range of 2.5–20 nM (Fig. 5I and J). The fitting equation is *y* = 977*x* + 39 965 (*R*^2^ = 0.9982), and the calculated LOD is 0.205 nM. The RSD values for intraday, interday repeatability, and 8-day stability were 1.11%, 0.93%, and 1.65%, respectively (Fig. 5K and L). The above results indicate that the constructed NIR/GSH dual-activated CRISPR–Cas13a biosensing platform has excellent linear response ability, repeatability, and stability for all three target miRNAs and that its detection sensitivity is superior to that of many reported miRNA detection methods (Table 1).

**Table 1:** Performance comparison of miRNA detection methods

Material	Time (min)	Amplification strategy	Linear range (nM)	Detection limit (nM)	Ref.
DNA	160	APE1/CHA	2.5–40	0.22	36
AuNP-FAM	120	DSN	0.3–8	0.3	37
T7 RNA	60	RCT	0–10	0.073	38
MNPs	110	ALP	0.1–25	0.08	39
DNA	130	DSN	1.2–3	0.36	40
UCNP/CRISPR–Cas13a/DNA	120	Cas13a/SDA	0.25–40	0.0042	This work
			2.5–20	0.205	

**Fig. 6:**
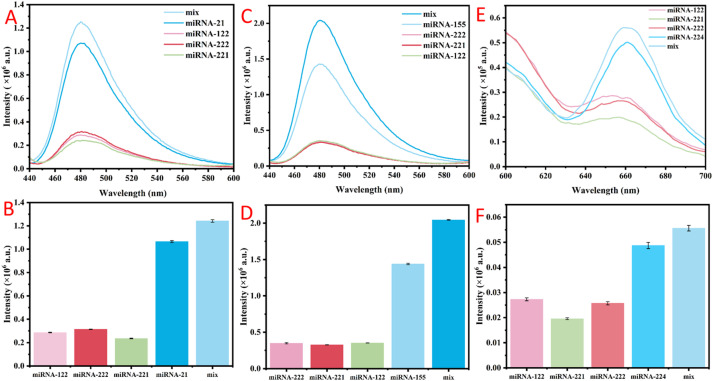
Evaluation of miRNA-21 (A and B), miRNA-155 (C and D) and miRNA-224 (E and F) detection specificity by a sensing platform in the presence of the target (100 nM) and the interfering substance (300 nM); *n* = 3.

Due to the relatively short length of miRNA sequences and minimal nucleotide differences between different miRNAs, the specific recognition ability of the constructed UCNP@mSiO_2_–Cas13a@Sp-H biosensor for miRNA-155, miRNA-21, and miRNA-224 is crucial. To evaluate its specificity, three other miRNAs (miRNA-122, miRNA-221, and miRNA-222) were selected as interference sequences, and their concentrations were increased to four times greater than that of the target miRNA (3 nM) to better verify the specificity of the sensor. At the same time, a mixed group containing three interfering miRNAs and three target miRNAs was set up to simulate the detection performance under multiple interference conditions. As shown in Fig. 6A, the fluorescence intensity of the experimental group with added interfering miRNA only slightly increased compared with that of the blank group, while the fluorescence intensity of the experimental group with added target miRNA-155 (Fig. 6A and B), miRNA-21 (Fig. 6C and D), and miRNA-224 (Fig. 6E and F) recovered significantly and that of the interference group was negligible. In addition, the fluorescence intensity of the mixed group also significantly increased. These findings demonstrate that the prepared UCNP@mSiO_2_–Cas13a@Sp-H biosensor has good specificity and can achieve stable and specific recognition and detection of miRNA-155, miRNA-21, and miRNA-224 in complex physiological environments.

### 3.5 Actual sample analysis

The applicability and reliability of the proposed UCNP@mSiO_2_–Cas13a@Sp-H biosensor were evaluated by detecting miRNA-155, miRNA-21 and miRNA-224 in human serum samples. Here, serum was obtained from healthy individuals, and experiments were conducted using standard addition methods. The test results are presented in Table 2, and the spiked recovery rate in the actual samples was between 98.97% and 109.24%, with a relative standard deviation (RSD, *n* = 3) of less than 4.613%. These findings demonstrate that the developed sensing system is capable of detecting miRNA-155, miRNA-21, and miRNA-224 in human serum samples, can adapt to complex body fluid environments, and can obtain accurate results.

**Table 2:** Analysis of miRNAs in serum samples

Sample	Added (nM)	miRNA-155			miRNA-21			miRNA-224		
		Found (nM)	Recovery (%)	RSD (%)	Found (nM)	Recovery (%)	RSD (%)	Found (nM)	Recovery (%)	RSD (%)
Serum1	2.5	2.682	107.28	4.362	2.363	94.52	4.613	2.624	104.96	4.23
	5	5.366	107.32	4.585	5.166	103.32	2.594	5.196	103.92	2.155
	7.5	7.742	110.6	2.169	7.682	102.46	3.359	7.582	101.09	3.666
Serum2	2.5	2.655	106.2	1.295	2.428	106.2	2.718	2.731	109.24	0.439
	5	5.284	105.68	2.971	5.261	105.68	2.064	5.184	103.68	2.45
	7.5	7.855	104.73	1.871	7.652	101.67	2.6	7.655	102.07	4.14
Serum3	2.5	2.642	105.68	3.823	2.442	97.68	0.491	2.542	101.68	3.698
	5	5.324	106.48	3.944	5.224	104.48	2.891	5.123	102.66	2.909
	7.5	7.423	98.97	0.35	7.462	99.49	4.369	7.432	99.09	3.045

## 4 Conclusion

In this work, an NIR/GSH dual-activated CRISPR–Cas13a biosensing platform was constructed on the basis of the UV-induced cleavage of photosensitive chemical bonds generated by UCNP stimulated by NIR light, as well as the mechanism of GSH reduction and disulfide bond cleavage, resulting in highly sensitive and specific determination of miRNA-155, miRNA-21, and miRNA-224. The LODs of the sensor for the three miRNAs mentioned above are 4.2 pM, 5.7 pM, and 0.205 nM. The spiked recovery rate in actual sample detection is 98.97–109.24%, demonstrating good analytical reliability. This sensing platform has the following outstanding advantages: (1) when UCNP is used to convert NIR light into ultraviolet light, it can accurately lyse pc linkers, and the background interference caused by NIR excitation is low; (2) the dual-activation strategy of GSH-responsive carrier degradation and NIR photocontrolled lysis significantly reduces nonspecific signals; (3) by utilizing the specific recognition and *trans*-cleavage activity of the CRISPR–Cas13a system, the high specificity of detection is improved; and (4) the ability to simultaneously detect multiple miRNA markers enhances the comprehensiveness and accuracy of diagnosis. In summary, the sensor achieves high-performance detection of disease-related miRNAs through the collaborative design of “dual activation control”, “CRISPR–Cas13a-specific recognition”, and “multitarget parallel detection”, providing a new method with good translational potential for early and noninvasive diagnosis of hepatocellular carcinoma.

## Ethical statement

All experiments were performed in accordance with the Guidelines of Zhangzhou Affiliated Hospital of Fujian Medical University, and experiments were approved by the ethics committee at Zhangzhou Affiliated Hospital of Fujian Medical University. Informed consents were obtained from human participants of this study.

## Conflicts of interest

The authors declare that they have no known competing financial interests or personal relationships that could have appeared to influence the work reported in this paper.

## Data Availability

The authors confirm that the data supporting the findings of this study are available within the article.
